# The role of surface EMG in predicting responsiveness of muscles to FES therapy after cervical SCI

**DOI:** 10.1186/s12984-025-01757-y

**Published:** 2025-11-07

**Authors:** Guijin Li, Gustavo Balbinot, Sharmini Atputharaj, Gita Gholamrezaei, Julio C. Furlan, Sukhvinder Kalsi-Ryan, José Zariffa

**Affiliations:** 1 KITE Research Institute, University Health Network, Toronto, Canada; 2 Institute of Biomedical Engineering, University of Toronto, Toronto, Canada; 3 Department of Biomedical Physiology and Kinesiology, Simon Fraser University, Burnaby, Canada; 4 Institute for Neuroscience and Neurotechnology, Simon Fraser University, Burnaby, Canada; 5 Rehabilitation Sciences Institute, University of Toronto, Toronto, Canada; 6 Department of Medicine, Division of Physical Medicine and Rehabilitation, University of Toronto, Toronto, Canada; 7 Division of Physical Medicine and Rehabilitation, Toronto Rehabilitation Institute, University Health Network, Toronto, Canada; 8 Institute of Medical Sciences, University of Toronto, Toronto, Canada; 9 Department of Physical Therapy, University of Toronto, Toronto, Canada; 10 Edward S. Rogers Sr. Department of Electrical and Computer Engineering, University of Toronto, Toronto, Canada

**Keywords:** Surface electromyography, Spinal cord injury, Machine learning, Recovery prediction

## Abstract

**Introduction:**

Cervical spinal cord injury (SCI) can severely impair upper extremity (UE) functions, limiting independence and quality of life. Prior clinical trials showed that functional electrical stimulation (FES) therapy can reduce UE impairment. However, the response to FES therapy is not consistent across all treated myotomes. Our objective was to predict the muscle response to FES therapy using electrophysiological biomarkers from baseline surface electromyography (sEMG) signals, in order to support treatment decisions at the point of care.

**Methods:**

We recruited 17 participants with cervical SCI, who were about to undergo FES therapy. Target UE muscles were identified for each participant by treating therapists. Baseline sEMG signals were recorded from the target muscles during resting and maximal voluntary contractions. Time- and frequency-domain features were extracted. The manual muscle testing (MMT) score was tracked through the therapy cycle, and used to categorize each muscle as a responder or non-responder. We explored classifiers including support vector machines, k-nearest neighbors, random forest, and logistic regression, with leave-one-participant-out cross validation. Models were trained on sEMG features alone, on clinical variables alone, and combinations of both.

**Results:**

The final dataset consisted of sEMG recordings of 132 muscles from 17 participants, and 33% of the muscles were considered responders. A Random Forest classifier with a forward-selected feature set yielded the best performance (Matthews correlation coefficient = 0.41, F1 score = 0.68, accuracy = 76%, precision = 0.72, recall = 0.42, and true negative rate = 0.92). With patient stratification based on motor completeness (AIS A-B vs. C-D), the model performance further improved. Included signal features were slope sign changes, mean and median frequency, and second-order spectral moment.

**Conclusions:**

Our results suggest that baseline sEMG signals combined with machine learning models may be used to predict muscle response to FES therapy in individuals with cervical SCI. The models were trained on a small and unbalanced sample and can be optimized with more participants in the future. This work contributes to improving the level of personalization and efficacy of FES therapy, and ultimately improve quality of life after SCI.

## Introduction

Spinal cord injuries (SCI) commonly result in neurological impairments and disability, disrupting the motor pathways between the brain and muscles. Cervical SCI can severely impair upper extremity (UE) function, which plays a crucial role in determining post-injury independence and quality of life. For individuals with cervical SCI, restoring upper limb function is the top priority [1, 2]. Comprehensive assessment of the neurological deficits related to SCI is essential for long-term prognostication and planning individualized treatment. The International Standards for Neurological Classification of Spinal Cord Injury (ISNCSCI) is a widely used clinical assessment for classification and longitudinal evaluation of individuals living with SCI [3]. Within ISNCSCI, motor scores from manual muscle testing (MMT) reflect muscle strength, the neurological level of injury (NLI) reflects the sensory and motor levels of an injury, and the American Spinal Injury Association Impairment Scale (AIS) assesses the severity of the SCI.

Numerous interventions have been developed to mitigate secondary medical conditions after SCI and to promote restoration of function [4, 5]. In recent years, the field has seen significant advancements in neuromodulation techniques, including epidural stimulation, spinal cord transcutaneous stimulation, and neuromuscular stimulation (NMES). Functional electrical stimulation (FES), a subtype of NMES, is one of the primary forms of peripheral neuromodulation [6]. It delivers short electrical pulses to contract paretic muscles. FES is characterized by its facilitation of *functionally useful* movements and emphasis on volitional movement attempts from the patients. Given preserved connections across the lesion and surviving lower motor neurons (LMN), FES may help strengthen damaged supraspinal pathways.

Over the past decades, FES therapy has shown significant potential in improving UE function [7, 8]. Typically, the therapy begins with the voluntary movement attempts by the patient, followed by the physiotherapist activating the stimulation to provide necessary support. Even a single session of assisted grasping and releasing has shown immediate positive effects in improving hand function of individuals with cervical SCI [9]. Long-term therapy (20–40 sessions) has demonstrated superior outcomes in enhancing upper limb functions compared to conventional therapies for individuals with sub-acute or chronic cervical SCI, even in motor complete SCI cases (AIS A and B) [7, 10–13].

Despite its great potential, FES therapy has had limited use in spinal cord rehabilitation due to several challenges. First, muscle response to the FES therapy varies due to the complex nature of SCI, as each case differs in specific injury location, preserved fibers across the injury zone, the integrity of LMN at or near the injury zone, and the remaining nerve fiber innervation [14–16]. Second, FES itself also has its limitations, such as the reversed recruitment order of motor neurons compared to the physiological process [17]. Additionally, FES therapy requires specialized clinician training and is often accessible only at specialized clinics, increasing financial and time-related burdens [18].

Among these challenges, the variability in patient outcomes is the greatest barrier to broader clinical adoptions, highlighting the need for predictive methods to tailor the therapy to individual patients. Predictive models to identify responder and non-responder muscles can help optimize treatment plans, improving time and medical resources allocation and patient outcomes. To the best of our knowledge, such methods are not available to predict muscle response to FES therapy.

With increasing availability of large SCI datasets (e.g., European Multicenter Study about Spinal Cord Injury [19], Rick Hansen spinal cord injury registry [20], and Japanese National Spinal Cord Injury Database [21]), data-driven prediction of SCI recovery has become feasible. Clinical metrics from ISNCSCI are often used in these prediction models, given its availability as the gold standard for assessments [22–24]. Electrophysiological assessments such as motor or somatosensory evoked potentials (MEP, SSEP) and nerve conduction studies (NCS) provide information about the integrity of neural pathways [25]. They have been shown to improve the prediction of functional improvement when combined with non-neurophysiological variables (sex, and ISNCSCI motor and sensory scores) [26]. However, these models were not intended to predict the responsiveness to FES therapy in the rehabilitation of individuals living with SCI.

Surface electromyography (sEMG) is a non-invasive measure of muscle activity that could collect data relevant to the predictive modeling of responses to FES therapy. Compared with other clinical assessments, sEMG is more sensitive in assessing motor recovery after SCI [27–31]. Even without visible muscle contractions, sEMG can detect underlying muscle activity during voluntary movement attempts. Despite its potential in capturing the impact from SCI, sEMG has not been fully utilized in clinical assessments and neurorehabilitation [32–37].

This study aimed to use baseline sEMG signals from UE muscles to predict response to FES therapy. With point-of-care tools based on pre-treatment sEMG combined with machine learning models, therapists could plan personalized treatment and improve FES therapy effectiveness after SCI. We hypothesized that sEMG-based predictive models can differentiate responder and non-responder target muscles at baseline, enabling personalized clinical treatment planning and ultimately improved patient outcome.

## Methods

**Fig. 1:**
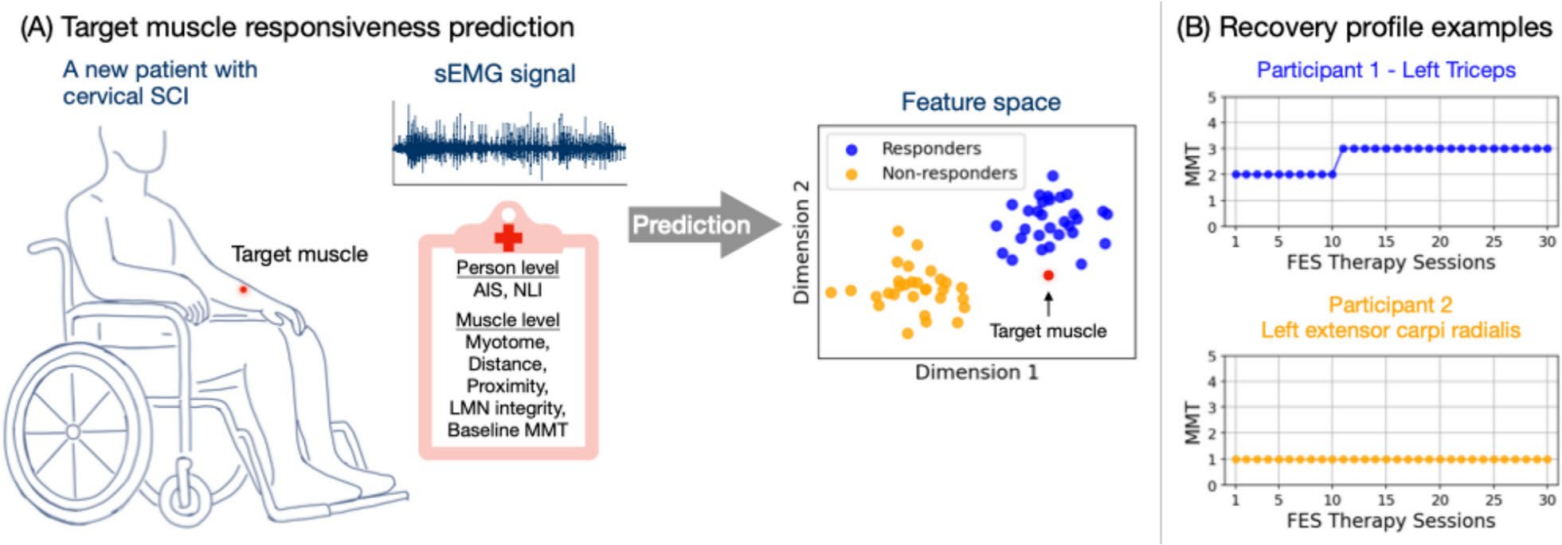
Conceptual study overview. **A** Prediction of a target muscle response to an upcoming FES therapy, using information extracted from sEMG signals and clinical variables. For demonstration purposes, the feature space is synthetic. **B** Recovery profile examples from two muscles after 30 sessions of FES therapy. Blue denotes responder muscles, and yellow non-responder muscles

We collected baseline sEMG data in individuals about to start FES therapy, and then tracked the strength recovery of individual muscles over the course of the therapy. Based on these recovery profiles, muscles were categorized as responders or non-responders. We then developed classifiers to predict the response of each to FES therapy, based on the sEMG and clinical information collected before the start of therapy. Figure 1 provides a conceptual overview of the intended use case of the classifier, and the individual steps are detailed in the following sections.

### Data collection

Data for this study was obtained from adult individuals with cervical SCI who were about to undergo FES therapy through one of three mechanisms at the Toronto Rehabilitation Institute, University Health Network: a clinic at our institution, a clinical trial of MyndMove therapy (Clinicaltrials.gov: NCT 03439319) [38], and directly recruited for this study (Clinicaltrials.gov: NCT 05462925). Approval was granted by the Research Ethics Board of the University Health Network (REB approval number: 19-5395.6). Written informed consent was obtained from all participants, and data stored on a secure hospital server. The FES therapy was delivered using the same methodology for all participants, regardless of the avenue of recruitment.

After the initial screening of each participant, the treating therapist identified for analysis 4 to 10 UE target muscles relevant to the FES therapy plan, which was based on available clinical information and the patient’s functional goals. The full list of muscles included in the dataset, combined across all participants, is provided in Appendix A.

We then recorded baseline sEMG signals individually from the target muscles per participant. The Bagnoli data acquisition system (Delsys, USA) was used with a sampling frequency of 4 kHz and bandpass filtering between 20 and 450 Hz. sEMG recordings were obtained during resting (one minute) and maximal (5 s) voluntary movement (three trials each). Resistance was provided according to the MMT protocol in the ISNCSCI or the Graded and Redefined Assessment of Strength, Sensibility, and Prehension (GRASSP).

Prior to the first FES session, clinical information and intramuscular EMG (iEMG) signal were also recorded. Participant-level clinical variables included months post injury, AIS grade and NLI. Muscle-level variables included myotome level, distance from myotome to NLI, MMT, and a customized variable Proximity. Myotome refers to the spinal cord segment primarily responsible for innervating a given muscle, based on the ISNCSCI. The Proximity variable quantified the anatomical proximity of a target muscle on a scale from 1 to 4, corresponding to shoulder, elbow, wrist, and finger movements, respectively (see Appendix A).

iEMG signals were recorded and analyzed by a specialized neurologist (JF) from a subset of the target muscles to provide supplementary information. From each muscle, iEMG signal was obtained using monopolar needle, following the current standards in clinical practice. The localization of the needle insertion points are standardized [39]. During muscle relaxation, the insertional and spontaneous activity were recorded in four directions corresponding to cardinal points in each muscle. When the participant initiated gradually increasing muscle contraction, the morphology, recruitment, and activation of the motor unit action potentials were recorded in each muscle. Based on the interpretation of the latter, a LMN injury was identified when the recordings showed a neuropathic pattern and reduced motor unit recruitment [40]. Given the complexity of the assessment and scheduling demands for specialized personnel, iEMG could only be recorded from a subset of muscles per participant. As a result, it is used here as supplementary information. In contrast, we are interested here in the predictive potential of sEMG due to its greater accessibility.

After baseline recordings, participants received between 11 and 40 one-hour sessions of FES therapy for upper limb function (mean number of sessions: 29.9 ± 7.6). The FES therapy was delivered using the built-in protocols of the MyndMove FES stimulator (MyndTec Inc., Mississauga, Canada), with the choice of protocols selected for each patient by the treating therapist. Before each session, the therapist performed MMT to assess strength of each target muscle. The strength change was tracked during the entire therapy cycle to establish a muscle recovery profile for each target muscle.

Twenty-two participants (184 muscles) underwent baseline sEMG assessments before starting FES therapy. Seventeen participants (136 muscles) finished the therapy cycle. Four muscles had an initial MMT score of the maximum value (5) hence were excluded from the analysis. As a result, 132 muscles from 17 participants were included in the data analysis. Table 1 summarizes the demographic information. The resulting study sample included individuals with different levels and severities of cervical injuries, including “discomplete” SCI (in which case no visible muscle contraction is observed yet EMG signal above noise level can be detected) [14, 27, 31, 41]. The iEMG data were recorded from 39 muscles from 12 participants. Each muscle was categorized into one of the three categories (Table 2): “no activation”, “damaged LMN”, or “inconclusive”.

**Table 1:** Baseline demographic information

Age (years, mean ± SD)	54.5 ± 16.3						
Sex	16 M, 1F						
Time post-SCI (months, mean ± SD)	34.2 ± 47.8						
Cause of SCI	15 Traumatic, 2 non-traumatic						
AIS	A	B	C	D			
Participant #	2	5	2	8			
Muscle # (n = 132)	16	41	16	59			
NLI	C2	C3	C4	C5	C6		
Participant #	1	5	7	2	2		
Muscle # (n = 132)	7	40	58	14	13		
Myotome	C5	C6	C7	C8			
Muscle # (n = 132)	18	48	20	46			
Distance (= Myotome-NLI)	0	1	2	3	4	5	6
Muscle # (n = 132)	5	11	43	32	29	9	3
Baseline MMT	0	1	2	3	4	5	
Muscle # (n = 136)	16	33	21	31	31	4 (excluded)	

*NLI* neurological level of injury, *Distance* myotome distance to the injury level, *MMT* manual muscle testing; number of samples available to each clinical variable is included in parenthesis

**Table 2:** LMN Integrity based on iEMG data

Category	Definition	MG # (n = 39)
No Activation	No voluntary muscle activation observed	8
Damaged LMN	Presence of LMN injury (accompanied by reduced recruitment)	14
Inconclusive	Muscle activation observed but no conclusive evidence for LMN injury	17

*LMN* lower motor neuron, *MG* muscle

### Responder identification

Accurate responder identification is crucial for effective model training. Here, responder muscles were defined by improvement in muscle strength (MMT scores) over the entire course of FES therapy (i.e., from the first to the last session). To ensure accurate and consistent evaluation of the trend in MMT scores over time, we quantified the change in MMT scores using Kendall’s tau rank correlation ($\alpha =0.01$) between scores and session numbers. The coefficient $\tau$ ranges from -1 (perfect disagreement) to 1 (perfect agreement). A muscle is identified as a responder if $\tau$ is positive with $p<\alpha$, indicating a statistically reliable positive association between MMT and session number. A positive Kendall’s $\tau$ necessarily indicates at least one ≥ 1-grade increase, since $\tau$ cannot be positive in the absence of any increases, and provides a mode stable indication of consistent improvement in comparison to a change between two discrete time points.

### Feature sets

From the 132 muscles, we obtained a total of 398 contraction trials (3 to 4 MVC attempts). We preprocessed the data using offset removal, filtering to reduce influence from motion artifacts (hardware bandpass filter between 20 and 450 Hz) and power line interference (60 Hz notch filter). The preprocessed data was then segmented. For each trial, we extracted one 3-s steady-state segment, for feature extraction. The steady-state segment was identified by the lowest standard deviation.

The initial feature list (“Full”, 24 features) was based on application of sEMG in myoelectric pattern recognition for prosthetic control [42–47]. (See Table B1 in Appendix B for formulas.) These include a broad range of features from time and frequency domain. Amplitude-based features peak-to-peak amplitude (p2p), mean absolute values (MAV), root mean square (RMS), and variance (VAR), and their extensions, difference variance version (DVARV), and difference absolute mean value (DAMV), generally reflect signal amplitude and energy content. Log-detector (logD) reflects exerted muscle force and is positively related to sEMG amplitudes [43, 45, 46]. Zero crossings (ZERC), slope sign changes (SSC), waveform length (wLen), Willison amplitude (wAmp), and second-order spectral moment (M2) are also time domain features with high computational efficiency, yet reflecting frequency domain properties such as motor unit firing [46–48, 50]. EMG histogram (EMGH), an extension of ZERC and wAmp, describes how often the signal reaches various amplitude levels [43, 46]. Mean and median frequencies (MeanF, MedF) directly reflect the frequency content of the signal and provide insights into fiber type composition and muscle fatigue. Autoregression coefficients (ARCO1-ARCO4) relate to the muscle contraction state, and cepstrum coefficients (Ceps1-Ceps4) reflect the rate of change in different frequency spectrum bands of the signal [43, 44]. Lastly, cardinality (Card) is the number of unique samples in the signal and has been shown to be highly descriptive of myoelectric activity [47].

Three approaches were used to curate sEMG feature sets from the Full list, aiming to reduce redundancy and dimensionality. The first approach identified the well-established Hudgins set (Hudgins) from the myoelectric control literature. The second approach is based on findings from a recent SCI sEMG modeling study [48], and identified features (MD set) that differentiate between upper and lower motor neuron damage and muscle fiber loss following SCI. The third approach applied principal component analysis (PCA) and forward feature selection (FWD) on the Full sEMG feature set to reduce its dimensionality. These methods yield four curated sEMG feature set, summarized below.

1. Hudgins: MAV, ZERC, SSC, wLen
2. MD: ZERC, SSC, wAmp, MedF, EMGH, ARCO3
3. PCA: Full set with PCA (with 90% variance explained)
4. FWD: Full set with forward selection

### Classification analysis and evaluations

We developed classification models using a distance-based classifier (k-nearest neighbors, KNN), tree-based classifiers (random forest, RF, and gradient boosting, GB), and a margin-based classifier (support vector machine, SVM). These classifiers represent a diverse range of approaches. The models were trained on the sEMG feature sets (Section C) with leave-one-participant-out (LOPO) cross-validation. Given the small dataset and the intrinsic variability of voluntary contractions, all trials for each target muscle were used. The mode of predicted labels across all trials was used to assign a final label.

We performed limited hyperparameter tuning to reduce overfitting risk in this small dataset. For KNN, we evaluated k from 2 to 7 using both Euclidean distance and cosine similarity, with distance-based weighting to break ties. For the tree-based classifiers, we tested with 100 and 200 estimators. For SVM, we tested both linear and Gaussian kernels. For each classifier, the configuration yielding the best performance was selected.

#### Performance metrics

The classification performance was primarily evaluated on the Matthews correlation coefficient (MCC, Eq. 1), which provides a balanced assessment by considering both classes. MCC ranges from -1 (complete disagreement) to 1 (perfect prediction), with 0 indicates no improvement from random guessing.

1 $$MCC= \frac{TP\times TN-FP\times FN}{\sqrt{(TP+FP)(TP+FN)(TN+FP)(TN+FN)}}$$

Additional metrics for *overall* and *participant-specific* prediction performance included: accuracy, precision, recall (true positive rate), specificity (true negative rate), and macro F1 score. For *overall* performance, metrics were calculated after aggregating predicted labels from all participants after LOPO cross-validation. For *participant-specific* performance, metrics were calculated on true and predicted labels of each participant separately during LOPO cross-validation.

Accuracy measures the proportion of correct predictions across both classes. Precision indicates the proportion of true responders among predicted positives. A classifier with high precision can reducing false positives and minimize the waste of time and resource related to the therapy. Recall (true positive rate, TPR) measures the classifier’s ability to correctly identify responders, which is crucial in this context. True negative rate (TNR) assesses a classifier’s ability to identify true negatives, ensuring that non-responders are not mistakenly classified as responders. High specificity helps avoid unnecessary treatments by reducing false positives. Macro F1 scores is the harmonized mean of recall and precision, averaged across both classes. It captures a balanced performance on both responder and non-responder classes.

#### Comparison using clinical variables

The sEMG model performance was compared to logistic regression models using clinical variables (Table 1). As mentioned earlier, participant-level clinical variables included months post injury, AIS grade and NLI, while muscle-level variables included myotome level, distance from myotome to NLI, baseline MMT score (prior to the first session), and proximity. Forward feature selection was applied to identify the most predictive variables. Top-performing clinical variables were combined with sEMG feature sets to assess their combined predictive effect.

#### AIS subgroup analysis

Since the models were trained on all participants (AIS A-D), we also evaluated classification performance specific to each AIS grade (A, B, C, and D). After obtaining predicted labels, we grouped these labels according to AIS grades and calculated performance metrics for each AIS group individually. This AIS grade-specific evaluation allowed us to evaluate how well the model trained on the full dataset generalized across different levels of motor completeness.

Additionally, we conducted a separate subgroup analysis by training models specifically on AIS A-B and AIS C-D data subsets. Unlike the AIS grade-specific evaluation described above, these subgroup-trained models only used a portion of the data, allowing us to evaluate classification performance for each motor completeness level, thereby assessing whether predictive performance differs between motor complete and motor incomplete injuries. The results may inform group-specific model adjustments during clinical implementation, ultimately enhancing clinical utility. Feature selection for these models followed the same process as for the full participant group (AIS A-D).

## Results

### Clinical variables

**Fig. 2:**
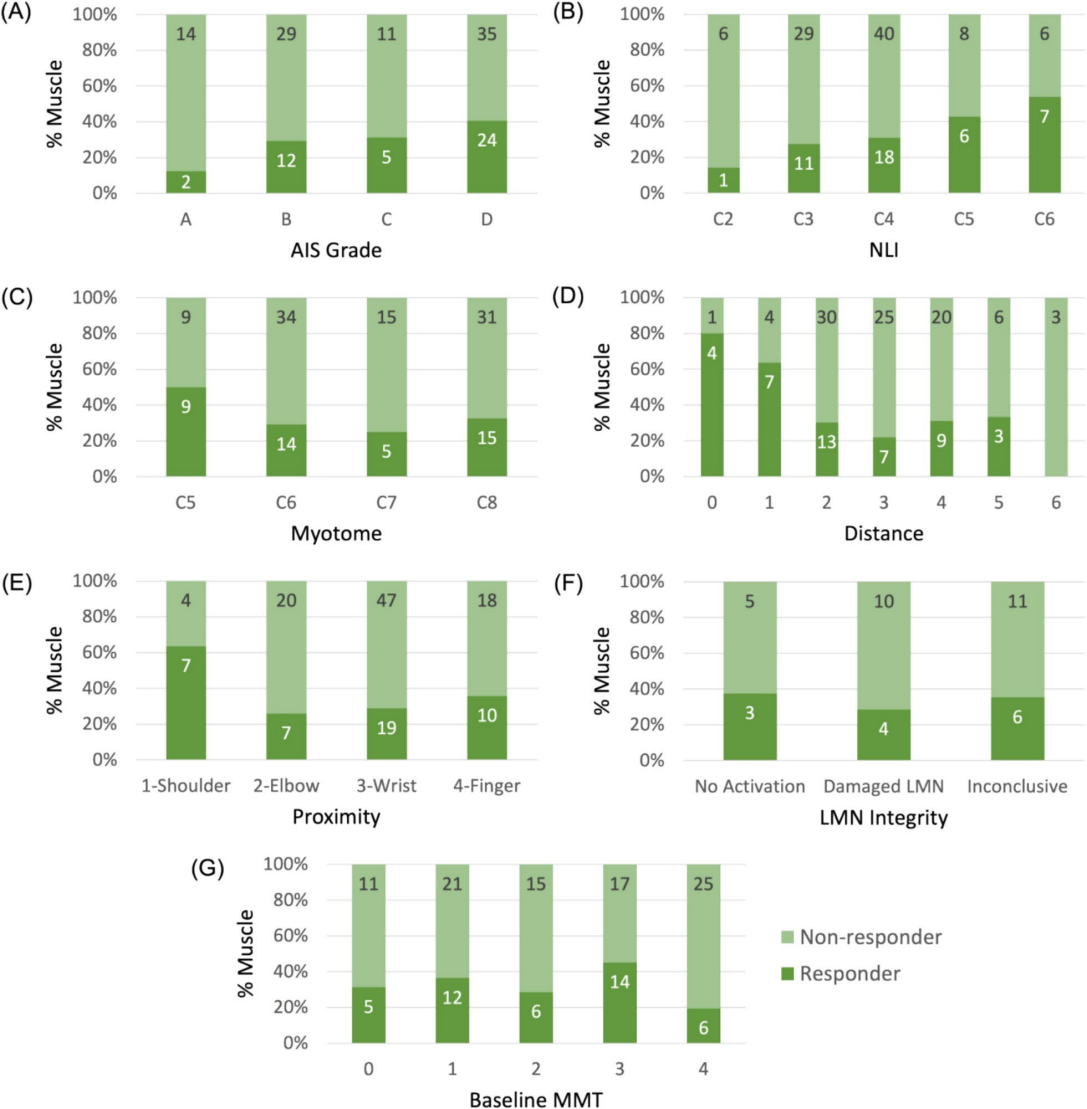
Distribution of responder (dark green) and non-responder (light green) muscles on selected clinical information: **A** AIS grade, **B** Neurological level of injury, **C** Myotome level, **D** Distance of myotome to the level of injury, **E** Proximity, **F** LMN integrity, and **F** Baseline MMT. Color proportion shows the respective percentage of muscles. Labeled numbers are the muscle counts

Among the 132 muscles (from 17 participants) included in the analysis, 43 (33%) were identified as responders based on MMT tracking. For AIS subgroups A-B and C-D, responders were 14 (25%) and 29 (39%), respectively. Figure 2 shows the distributions of responder and non-responders across the clinical variables. Most clinical variables, except for AIS grade and NLI, did not show consistent trends in predicting muscle responsiveness. Notably, as AIS grade progresses from A (sensory and motor complete) to D (sensory and motor incomplete), the percentage of responder muscles increased monotonically. A similar monotonic increase was observed as NLI shifts from C2 (higher cervical level) to C6 (lower level). However, while AIS grade and NLI reflect overall participant status, they cannot predict responsiveness at the individual muscle level, which is the primary focus of this analysis.

### Classification performance evaluation

Table 3 summarizes the *overall* performance of the best classifiers trained on each feature set either using only clinical variables or only sEMG features. For each set, four to six features were identified (details in the table). The top-performing classifier for each feature set was identified based on MCC, and a value around 0.26 to 0.41 indicate mostly modest performance. Given the imbalanced dataset, low TPR (0.33) and high TNR (0.67) are expected by chance. Combining clinical variables with sEMG features did not improve model performance so these results are not shown in Table 3; the highest MCC for these combinations was 0.24 from MD combined with clinical variables, with corresponding F1 score of 0.62 and accuracy of 66%.

**Table 3:** Prediction performance from the best classifiers and feature sets

	By chance	Clinical variables	sEMG feature sets			
Features	–	NLI, Proximity, AIS, Distance	*Hudgins* MAV, ZERC, SSC, wLen	*MD* ZERC, SSC, wAmp, MedF, EMGH, ARCO3	*PCA* First 4 PCs (92.7% variance explained)	*FWD* SSC, M2, MeanF, MedF
Classifier	–	Linear logistic regression	Linear SVM	Linear SVM	Linear SVM	RF
MCC	0	0.26	0.29	0.28	0.28	0.41
F1 score	0.50	0.57	0.64	0.63	0.63	0.68
Accuracy	56%	71%	71%	70%	70%	76%
Precision	0.33	0.69	0.59	0.57	0.57	0.72
Recall (TPR)	0.33	0.21	0.40	0.40	0.40	0.42
TNR	0.67	0.96	0.87	0.85	0.85	0.92

*NLI* neurological level of injury, *AIS* AIS grade, Distance = distance of myotome level below NLI, Features sets (Method-Section C): MD = MD set, PCA = Full set + PCA, FWD = Full set + Forward selection, SVM = support vector machines, RF = random forest classifier

In the clinical-only feature set, forward selection with linear logistic regression identified the combination of NLI, Proximity, AIS, and Distance as the best set, with an MCC of 0.26. This model achieved high TNR (0.96) and moderate precision (0.69), effectively identifying true non-responders and minimizing false positives. However, the low recall/TPR (0.21, lower than expected 0.33) indicates a high false-negative rate, meaning many true responders were classified as non-responders, likely influenced by the dataset’s imbalance.

Among the feature sets with only sEMG features, FWD set outperforms other feature sets, with the highest MCC (0.41), F1 score (0.68), accuracy (0.76), precision (0.72), recall (0.42), and TNR (0.92). This suggests that the FWD (with RF) is effective at distinguishing between responders and non-responders. However, the recall remains modest (0.42), suggesting that not all true responders are identified. The other three sEMG feature sets (Hudgins, MD, and PCA) each paired with a linear SVM classifier, have similar moderate performance across the metrics, with MCC between 0.28 and 0.29, and F1 score between 0.63 and 0.64. These sets have balanced precision and recall, but lower MCC and macro F1 score compared to FWD, highlighting the benefits of effective feature selection.

**Fig. C1:**
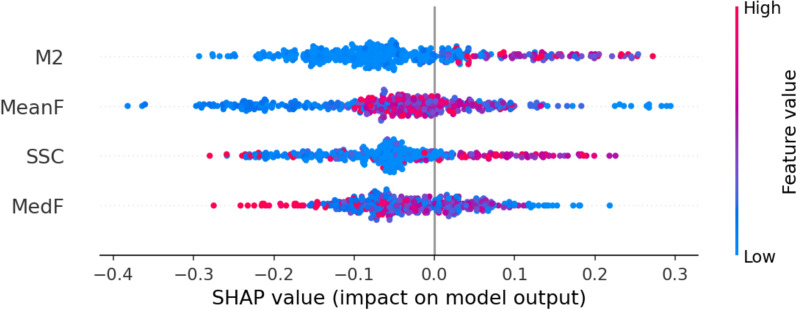
SHAP summary plot showing the contribution of each feature in the best-performing classifier (RF) and feature set (FWD) combination. Features are ordered by their mean absolute SHAP value (highest at the top). Each point represents one trial. Color indicates the magnitude of the feature value (red = high, blue = low). Positive SHAP values indicate contributions toward predicting a positive (responder) class

To further interpret the contribution of features in the FWD set (with RF), we applied SHapley Additive exPlanations (SHAP) to assess how each feature influenced model predictions. The SHAP summary plot (Appendix C, Figure C1) revealed that M2 was the most influential feature in the selected set, with higher values associated with higher predicted responsiveness. Since SHAP explains model output per sample, the results are based on trial-level predictions and do not directly reflect the final labels used in evaluation, which were the mode of predicted labels across all trials of a muscle.

**Fig. 3:**
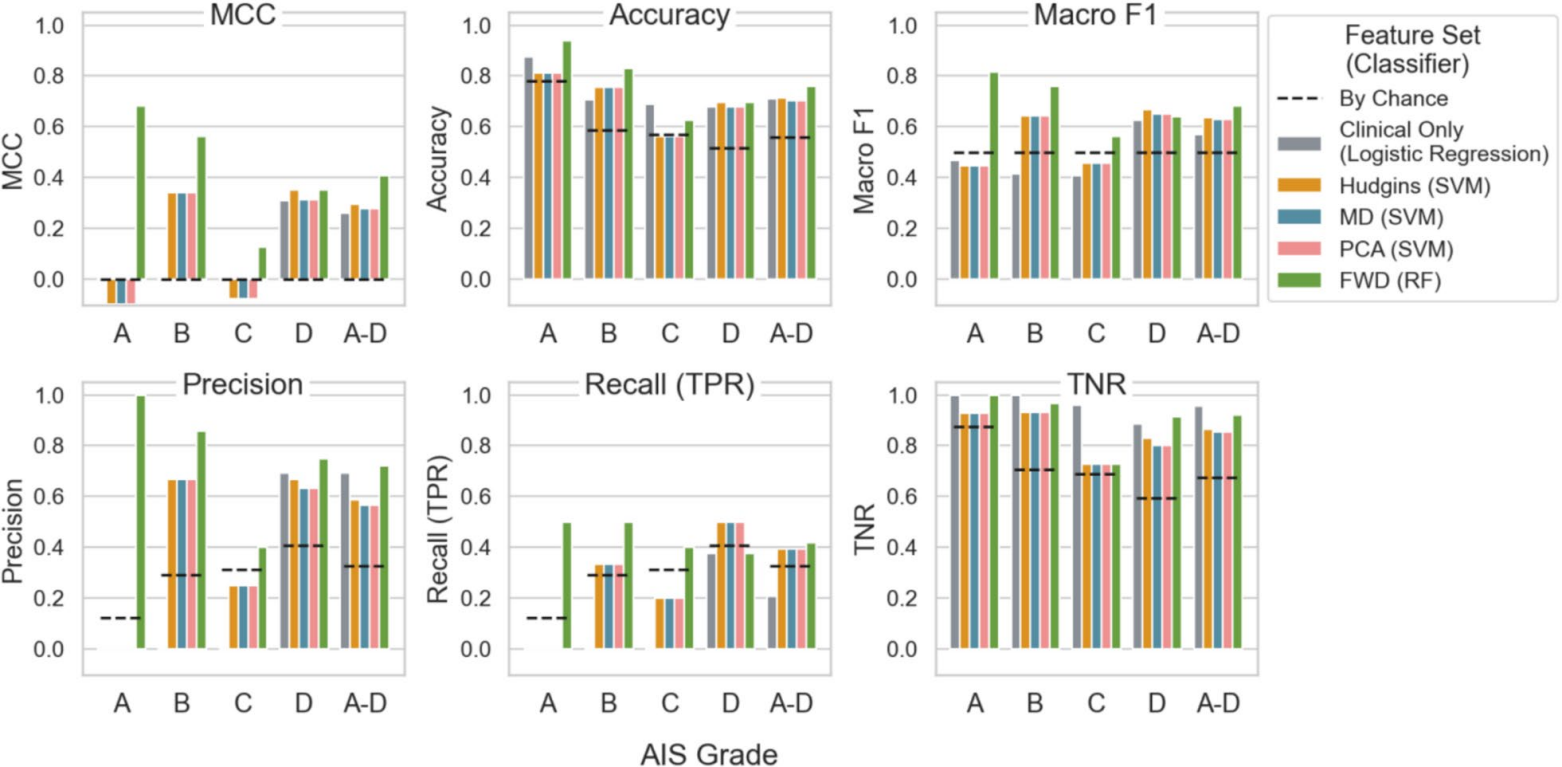
Overall performance of classifiers trained on all participants (AIS A-D) evaluated separately across AIS grades (**A**, **B**, **C**, and **D**). AIS A-D is plotted for reference. Each panel plots one evaluation metric: Matthews correlation coefficient (MCC), accuracy, macro F1, Precision, Recall (true positive rate, TPR), and true negative rate (TNR). Black dashed lines indicate metric values expected by chance. Each bar represents a feature set with the classifier used (Table 3): Clinical Only, Hudgins, MD, PCA, and FWD. SVM = support vector machines, RF = random forest

While Table 3 summarizes the overall performance of the winner classifier for each feature set, Fig. 3 shows how these classifiers performed when evaluated separately across AIS grades (A, B, C, and D). The FWD feature set consistently outperformed others, achieving higher values in MCC, accuracy, macro F1, precision and recall in most AIS grades. It is also the only feature set with MCC and macro F1 values above chance in all AIS grades. This suggests that FWD is the most effective feature set for distinguishing between responders and non-responders. In contrast, the Hudgins, MD, and PCA feature sets show similar and moderate results, generally performing above chance but below the FWD set. The Clinical Only feature set, while achieving high TNR, is less effective overall, with notably low MCC and recall scores. It suggests that clinical variables alone are insufficient for accurate prediction.

Classifier performance also varies notably across AIS grades, particularly in MCC, precision and recall. For AIS A and C, MCC scores are close to or below zero, suggesting that models (except for RF on FWD) trained on all participants perform no better than random guessing when evaluated on these groups. These grades also show lower precision and recall, indicating difficulty in accurately identifying true responders. In contrast, MCC scores are notably higher for AIS B and D, indicating better overall classification performance. This trend aligns with higher precision and recall scores in these groups, suggesting that the models trained with all participants can more effectively distinguish responders and non-responders in AIS B and D groups. The sensory preservation in AIS B and greater motor function in AIS D may contribute to the better classification performance evaluated in these groups. But more importantly, the dataset has more muscles from participants with AIS B and D, 41 and 59 respectively.

TNR is consistently high and above the by-chance level across all feature sets and AIS grades, which is expected given the imbalanced dataset with more non-responders than responders. However, recall (TPR) scores are generally lower, particularly in AIS A and C, highlighting a trade-off: while models are effective at identifying non-responders (high TNR), they struggle to identify true responders accurately (lower TPR). This imbalance affects overall model performance, as reflected by lower MCC and F1 scores, especially in AIS A and C.

**Fig. 4:**
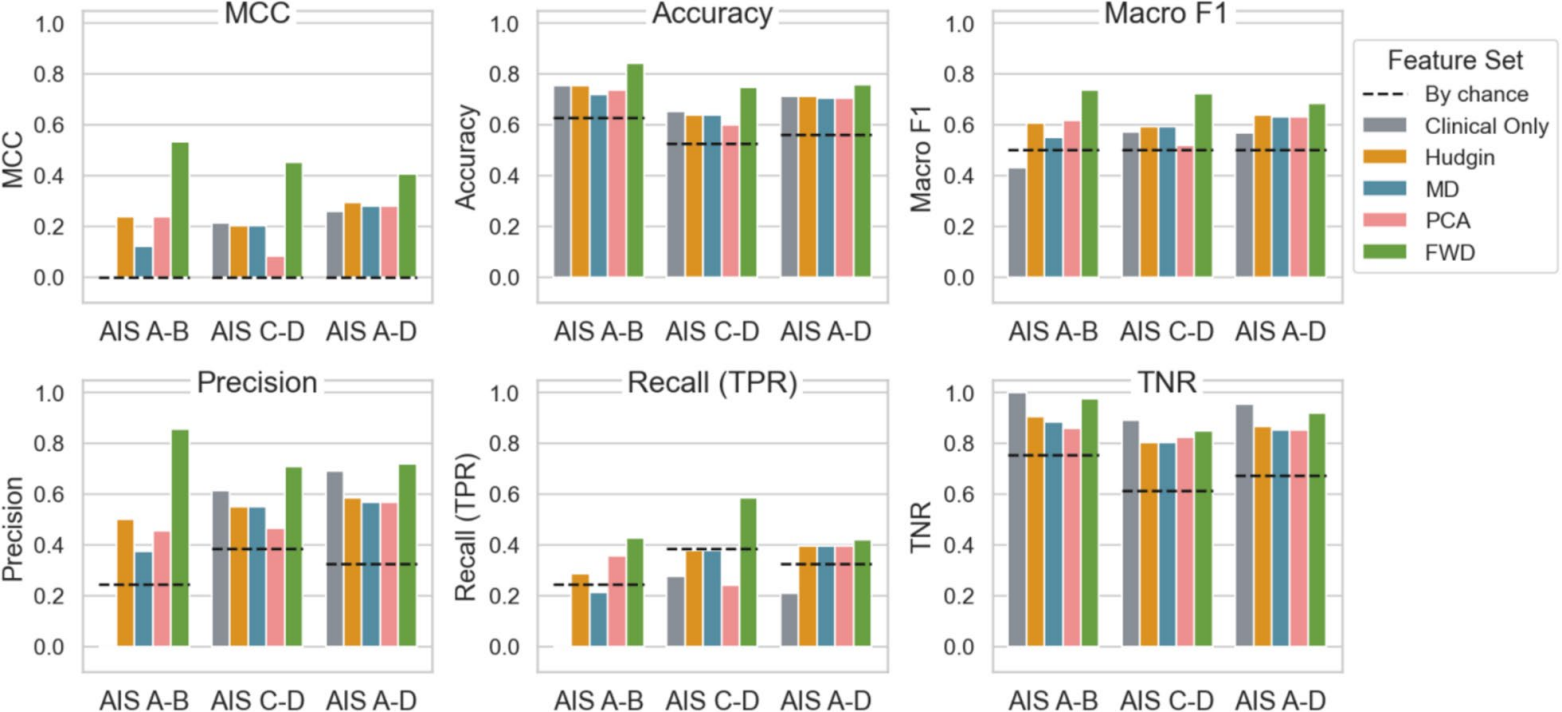
Overall performance of classifiers trained on all participants (AIS A-D) and on stratified AIS subgroups (AIS A-B, motor complete; AIS C-D, motor incomplete). Each panel represents one evaluation metric: Matthews correlation coefficient (MCC), accuracy, macro F1, Precision, Recall (true positive rate, TPR), and true negative rate (TNR). Black dashed lines indicate metric values expected by chance. Each bar represents a feature set (Table 3): Clinical Only, Hudgins, MD, PCA, and FWD

While Fig. 3 shows performance across AIS grades using models trained on all participants (AIS A-D), Fig. 4 compares the performance of classifiers trained on the AIS A-D and on stratified subgroups: motor complete (AIS A-B), and motor incomplete (AIS C-D). Training models on AIS subgroups generally improves classification performance, as indicated by increase in MCC, macro F1, and recall, compared to training on the entire dataset. The FWD feature set remains the strongest performer across all subgroups, with consistently higher values in MCC, accuracy, macro F1, precision, and recall. Note that the winning classifier with FWD is KNN for the subgroups (AIS A-B and AIS C-D). This suggests that the FWD feature set retains its effectiveness in distinguishing between responders and non-responders, even when models are trained on specific subgroups.

In contrast, the Hudgins, MD, and PCA feature sets exhibit moderate performance, generally above chance but consistently below the FWD set, regardless of the subgroup. The Clinical Only feature set shows high TNR but low MCC, precision and recall scores, particularly for AIS A-B. It reinforces that clinical variables alone are insufficient for accurate prediction, especially within the motor complete subgroup.

**Fig. 5:**
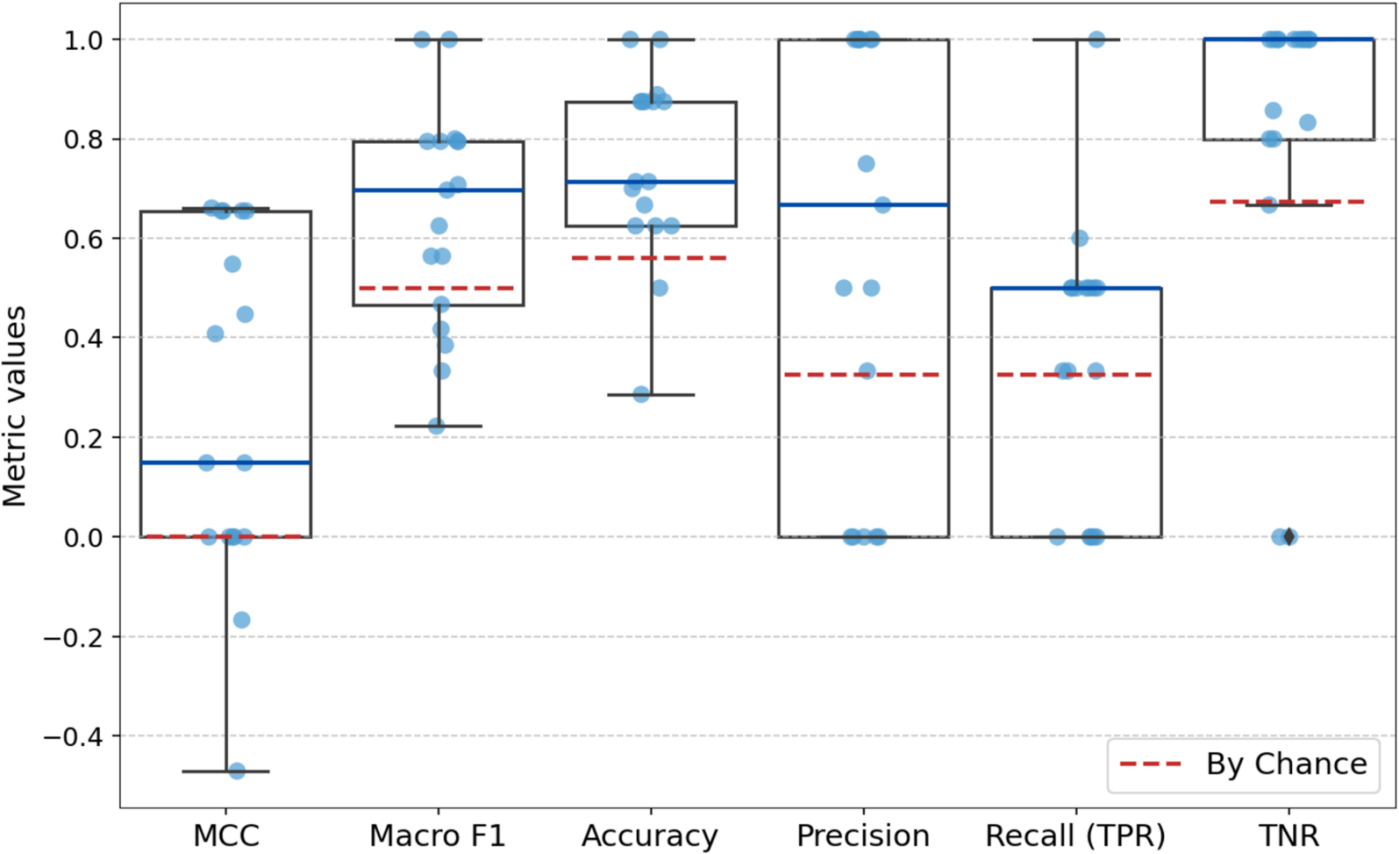
Participant-specific performance distribution of leave-one-participant-out cross-validation of the best classifier and feature set combination (Random Forest classifier on FWD). Evaluation metrics include Matthews correlation coefficient (MCC), macro F1, accuracy, precision, recall (true positive rate, TPR), and true negative rate (TNR). Blue lines of the boxplot highlight the median metric values. Red dashed lines indicate metric values expected by chance. Light blue dots are individual participants

Figure 5 illustrates the participant-specific LOPO cross validation performance of the best classifier (RF) and feature set (FWD) combination trained on the full dataset (AIS A-D). TNR has the highest mean, reflecting the model’s strong ability to correctly identify non-responders, which aligns with the class imbalance in the dataset. However, recall (TPR) shows moderate performance across participants, suggesting some difficulties in consistently identifying the true responders.

Among the metrics, accuracy has the lowest variability (mean ± SD = 0.75 ± 0.19), indicating the model’s overall correctness is relatively consistent across participants. In contrast, precision (0.57 ± 0.44) and MCC (0.26 ± 0.35) exhibit the greatest variability, indicating significant differences in model performance across participants. This variation likely reflects individual differences in muscle responses, with the model performing better in some participants than in others. In fact, among the 17 participants, two had no responder muscles, resulting recall of zero. Eight participants had 25% or less responder muscles, making it challenging to achieve high recall values, especially at the participant-specific level. However, in one exception, for the participant with recall of 1.0, the model successfully predicted the 1 responder (out of 8 target muscles).

## Discussion

The primary goal of this study was to predict muscle response to FES therapy from baseline sEMG signals for individuals with cervical SCI. sEMG has the potential to be integrated into point-of-care tools to provide biomarkers for clinical decision support and enable precision rehabilitation approaches. By knowing which muscles are likely to benefit, therapists can make informed decisions into the use of the limited therapy time available. We evaluated classification models trained on clinical variables alone, sEMG features alone, and combinations of both. Leave-one-participant-out (LOPO) cross validation was used to assess robustness across participants.

Our findings indicate that sEMG feature sets, particularly the FWD feature set (slope sign changes, mean and median frequencies, and M2), consistently outperform models using clinical variables alone. The FWD feature set combined with a random forest classifier achieved the highest performance across multiple metrics, including MCC (0.41), accuracy (0.76), and macro F1 (0.68). Combining clinical variables with sEMG features did not improve model performance, underscoring the unique predictive values of sEMG features. Additionally, training models on specific AIS subgroups (motor complete and motor incomplete) improved performance, particularly in AIS A-B, compared to models trained on the entire dataset.

### Limitations of clinical variables alone

Although the progression of AIS grade from A to D correlates with an increasing percentage of responders (Fig. 1), AIS was not identified as the single most important predictor in logistic regression with forward feature selection. Resulting clinical variables also include NLI, Proximity, and Distance. The logistic regression model trained on these variables demonstrated moderate performance (Table 2), with MCC, macro F1 score, accuracy, precision, and TNR all above expected values by chance. Recall (0.21) from the model is below the expected chance level (0.33), indicating the difficulty in correctly identify true responders. This low recall suggests that some muscles that could benefit from the FES therapy might be overlooked.

When evaluating the logistic regression model’s performance per AIS grade (Fig. 3), the limitations of relying solely on clinical variables become more evident, particularly for AIS A, B, and C, where MCC, precision, and recall are all zero. The poor performance on AIS B is especially notable, as it includes a larger number of participants compared to AIS A and C. Training the model on AIS subgroups slightly improved performance for the motor incomplete group (AIS C-D) but did not enhance results for motor complete group (AIS A-B). In fact, the model predicted all AIS A-B muscles to be non-responders, resulting in 14 (25%) false negatives. With no positive predictions, MCC, precision, and recall remained at zero. By relying on this model with only clinical variables, no muscles from patients with AIS A or B would be selected for FES therapy—or potentially for other treatment as well.

### Prediction with sEMG features

In contrast, models using sEMG features alone consistently outperformed those based on solely clinical variables, particularly the FWD set with RF. The FWD set achieved the highest values in all metrics, including MCC (0.41), accuracy (0.76), and macro F1 (0.68), though it did not achieve the highest TNR due to the class imbalance. When trained on all participants, FWD set was the only one to obtain above-chance performance across all AIS grades (Fig. 2), suggesting that it captures essential sEMG characteristics relevant to predicting the response to the FES therapy.

The FWD feature set (SSC, M2, mean and median frequencies) captures diverse aspects of motor unit firing and recruitment patterns by integrating both time- and frequency-domain information. This breadth is likely key to its strong predictive performance, as no single feature alone can fully characterize the neuromuscular output, especially after SCI. For example, M2, a time-domain feature characterizing frequency-domain behavior, quantifies the temporal variability of the signal by computing the squared difference between consecutive time samples. Higher M2 values may reflect more abrupt signal changes and complex activation patterns, which contributes towards predicting positive (responder) class in the SHAP analysis (Appendix C). SSC is related to the frequency of slope changes and indicative of motor unit firing irregularity. Mean and median frequencies, which summarize the distribution of spectral power, also contributed but with greater variability across samples. These findings highlight the physiological relevance of the selected features and support the use of diverse and broad feature sets to improve prediction robustness in the heterogeneous SCI population.

Moreover, Fig. 3 shows that training models specifically on motor completeness subgroups (AIS A-B vs. C-D) leads to further performance improvement for RF with the FWD set. This subgroup-specific training enhances MCC, accuracy, macro F1, precision, and TNR, particularly for AIS A-B, highlighting the advantages of tailoring models to motor completeness levels. This approach appears to capture more distinct sEMG patterns within each subgroup, allowing for improved classification performance. Given the small dataset, we did not separate subgroups further by individual AIS grade, though this may provide further improvements with a larger sample size.

### Impact of imbalanced dataset on recall (TPR) and TNR

The consistently high TNR across feature sets and models can be attributed to the dataset’s class imbalance, where non-responders are more frequent than responders. This imbalance leads to models that are effective in identifying non-responders but struggle with recall, particularly in AIS A and C, where MCC scores were close to or below zero (Fig. 2) for most models. This low recall indicates that while models perform well in identifying non-responders, they may overlook true responders, limiting the practical utility. Results from Fig. 4 suggests that subgroup-specific training partially alleviates this issue by improving recall within more homogeneous groups, especially in the motor incomplete subgroup, where the model appears better suited to capturing true responder characteristics.

### Variability across muscles and participants

Precision variability across participants from RF on the FWD set (Fig. 5) underscores the challenge of achieving consistent responder classification. Precision had the highest variability, followed by MCC, TNR and recall. This variability suggests that while the FWD feature set with RF generally performs well, individual differences in muscle response create inconsistencies in predicting true responders. Notably, the high variability in precision and MCC indicates that certain participants’ sEMG signals are easier to classify than others, possibly due to differences in baseline after SCI or other individual-specific factors. The results in Fig. 5 should however be interpreted with caution, considering the low number of muscles per participant that may impact the robustness of the metrics in this portion of the analysis.

We recognize that differences in muscle type and size may influence sEMG signal characteristic and classification outcomes. Although this variability was not stratified in the current analysis, future studies with more data may explore muscle-specific stratification approaches.

### In the context of existing literature

There exists intensive literature in predicting functional recovery after SCI [23, 24, 49]. However, to the best of our knowledge, no prior study exists to provide prediction of muscle response to FES therapy, a promising intervention for restoring motor function. Our study is the first attempt to address the gap in the literature and focuses on muscle-level prediction of FES therapy outcomes. While clinical variables such as AIS grade and NLI provide general prognostic information [22–24], our findings indicate that baseline sEMG features, particularly the FWD feature set, are more effective for predicting responses to FES therapy. Unlike available clinical information, sEMG captures neuromuscular activation patterns that reflects residual motor connectivity. The FWD feature set combined with a random forest classifier consistently outperformed models using clinical variables alone, suggesting that sEMG features capture unique, functionally relevant information at the muscle level. Notably, combining clinical variables with sEMG features did not enhance model performance, reinforcing the unique predictive value of sEMG alone.

Prior studies have shown that stratifying SCI patients into specific subgroups based on motor completeness or baseline neurological impairment can improve prognostic accuracy, such as the Unbiased Recursive Partitioning regression with Conditional Inference Trees (URP-CTREE) model [50]. The URP-CTREE model has been used to stratify patients with acute traumatic SCI into homogeneous subgroups to optimize recovery predictions and enhance the design of clinical trials. We explored training separate models for motor complete (AIS A-B) and motor incomplete (AIS C-D) groups. Our findings similarly suggest that subgroup-specific training improves classification performance, particularly in identifying responders, by allowing models to capture subgroup-specific sEMG patterns related to motor completeness.

### Choice of MMT as an outcome measure

MMT is a practical and commonly used clinical assessment for muscle strength and was used as the primary outcome measure. Because of its simplicity and accessibility in a clinical setting, MMT was administered before each FES therapy session to track the target muscle strength, without the need for additional scheduling. To ensure consistency across sessions and raters (therapists), we implemented standardized protocols from ISNCSCI and GRASSP.

While not feasible for frequent longitudinal data collection, other modalities such as imaging or motor evoked potentials could provide quantitative insights into muscle structure and corticospinal connectivity in response to FES therapy. Combining these tools with baseline sEMG could offer a comprehensive evaluation framework, capturing both functional and structural aspects of the recovery. A multi-modal approach with measurements before and after FES therapy or at multiple timepoints throughout the therapy cycle could help refine responder identifications and provide more accurate evaluation, enabling more robust predictive model development.

### Limitations and future directions

In this section, we discuss several limitations that should be considered when interpreting the findings and future research directions.

#### Expanding dataset diversity and demographics

First, there was only one female participant (less than 6%), which does not reflect the proportion seen in the SCI population [51] and restricts the study’s generalizability across sex. A more balanced sample would provide a clearer understanding of potential differences in response to FES therapy between male and female participants.

The dataset also has a higher number of non-responders (67%) than responders. This imbalance likely contributed to the high true negative rate (TNR) observed across models, as well as the relatively low recall, indicating that the models may be better at identifying non-responders than true responders. While we attempted to compensate for this effect by evaluating models with robust metrics such as MCC and F1 score, future studies with more balanced responder and non-responder groups would help validate these findings and improve the model’s sensitivity to true responders.

Overrepresentations of AIS D injuries (45%) and C3–C4 level of injury (74%) are also observed. AIS D often reflects more treatment options and better prognostics. Individuals with motor complete cases (AIS A or B) often face lower expected recovery potential. Our results show that clinical information alone typically predicts all muscles in AIS A cases to be non-responders, effectively closing the door to FES therapy for this subgroup. This exclusion is problematic, as AIS A patients represent a group in dire need of interventions. These imbalances hinder the generalizability of findings to the broader SCI population, which exhibits great demographic and clinical diversity.

While we obtained promising results by training models on specific AIS subgroups, the relatively small sample size prevented further stratification by individual AIS grades. Future studies with larger datasets may benefit from more granular subgroup analysis to capture subtle differences in muscle response within each AIS grade, potentially enhancing predictive accuracy.

Although our primary goal in this study was to explore generalizable predictive patterns in baseline sEMG across target muscles for FES therapy, a larger dataset would allow for investigation of the impact from muscle anatomical variability, including muscle type (e.g., biarticular vs uniarticular) and size. Along with sex and other person- and muscle-level variables, muscle type and size could be explored as predictive variables.

#### Beyond binary classification

In this study, binary classification results were used to evaluate model performance. With a larger dataset, future work could move beyond binary prediction to estimate changes in MMT scores directly. Predicting both the magnitude and the timing of MMT improvement could provide clinicians with more detailed guidance for treatment planning and help set realistic expectations for patients. Also, the confidence level of the prediction could be investigated to indicate the likelihood of responding, providing additional decision support to treating therapists beyond the current binary classification.

#### Integration of multi-channel perspectives

The experiment was designed with a specific clinical point-of-care implementation in mind: take sEMG measurements from a potential target muscle during voluntary contractions based on MMT protocols, and predict its responsiveness to FES therapy in real time or a short amount of time. As such, simplicity and clinical feasibility were the top priorities—a simple setup with bipolar electrodes and no posture restrictions, with only one recording session. Analysis was also done on individual muscles, instead of multiple muscles together.

While this approach aligns with the implementation goals, it limits the depth of electrophysiological insights. Incorporating signals from multiple channels to analyze agonist and antagonist interactions or co-activation patterns could be beneficial. In our experiments, firing of non-target muscles are often observed even though only the target muscle was voluntarily contracted. Compared to using information solely on the target muscle, these patterns could potentially provide more information regarding the systemic effect of SCI, leading to a more robust muscle-specific prediction.

## Conclusions

The results of our study suggest that machine learning models using sEMG signals can be used to predict the response to FES therapy in each myotome. With our dataset of 132 muscles from 17 participants, we found that muscle-specific responsiveness to FES therapy could be predicted more accurately using an sEMG feature set than using clinical variables alone. Participant stratification based on motor completeness (AIS A–B vs. C–D) further improved classifier performance. This study highlights baseline sEMG as a promising and non-invasive predictor for FES therapy response when combined with machine learning models, potentially enabling more personalized and effective rehabilitation strategies after SCI. In this manner, sEMG features may provide treating therapists with more specific insights into muscle response potential than currently available clinical measures, supporting more effective and targeted intervention planning in SCI rehabilitation.

## Data Availability

The datasets analyzed during the current study are available upon reasonable request to the authors and completion of a data sharing agreement.

## Appendix A

Upper extremity muscles identified as FES therapy targets (combined from all participants):

* denotes muscles with iEMG testing in at least one participant.

Shoulder muscles (proximity = 1): *anterior and middle deltoid muscles.

Elbow muscles (proximity = 2): *biceps brachii muscle, *triceps brachii muscle.

Wrist muscles (proximity = 3): *extensor carpi radialis longus muscle, extensor carpi ulnaris muscle, *flexor carpi radialis muscle.

Hand and finger muscles (proximity = 4): *flexor digitorum superficialis muscle, *extensor digitorum communis muscle, flexor pollicis longus and brevis muscles, *opponens pollicis muscle, *first dorsal interosseous muscle, and *abductor pollicis brevis muscle.

## Appendix B

See Table B1.

**Table B1:** sEMG feature abbreviations and formulas

sEMG features	Abbreviation	Formula $N$: total number of samples in the data segment $i$: $i$ th sample in the data segment
Mean absolute values	MAV	$MAV=\frac{1}{N}\sum_{i=1}^{N}\left|{x}_{i}\right|$
Difference variance version	DVARV	$DVARV=\frac{1}{N-2}\sum_{i=1}^{N-1}{\left({x}_{i+1}-{x}_{i}\right)}^{2}$
Second-order spectral moment	M2	$M2=\sum_{i=1}^{N-1}{\left({x}_{i+1}-{x}_{i}\right)}^{2}$
Difference absolute mean value	DAMV	$DAMV=\frac{1}{N-1}\sum_{i=1}^{N-1}\left|{x}_{i+1}-{x}_{i}\right|$
Waveform length	wLen	$wLen=\sum_{i=2}^{N}\left|\Delta {x}_{i}\right|, \Delta {x}_{i}={x}_{i}-{x}_{i-1}$
Zero crossings	ZERC	$\left\{\left({x}_{i}>0\wedge {x}_{i+1}<0\right) \vee \left({x}_{i}<0 \wedge {x}_{i+1}>0\right) \right\} \wedge \left|{x}_{i}-{x}_{i+1}\right|\ge \varepsilon , \varepsilon =0$
Slope sign changes	SSC	$\left\{\left({x}_{i}>{x}_{i-1}\wedge {x}_{i}>{x}_{i+1}\right) \vee \left({x}_{i}<{x}_{i-1}\wedge {x}_{i}<{x}_{i+1}\right) \right\} \wedge \left\{\left|{x}_{i}-{x}_{i+1}\right|\ge \varepsilon \wedge \left|{x}_{i}-{x}_{i-1}\right|\ge \varepsilon \right\},, \varepsilon =0$
Willison amplitude	wAmp	$$wAmp=\sum_{i=2}^{N}f(\left|{x}_{i}-{x}_{i-1}\right|),f\left(x\right)=\left\{\begin{array}{c}1, x>\varepsilon \\ 0, otherwise\end{array}\right., \varepsilon =0$$
Log-detector	logD	$logD={e}^{\frac{1}{N}\sum_{i=1}^{N}\text{log}\left|{x}_{i}\right|}$
Autoregression coefficients	ARCO $i$ (e.g., ARCO1)	${x}_{k}=\sum_{i=1}^{p}{a}_{i}{x}_{k-i}+{e}_{k},$ where ${a}_{i}=$ autoregressive coefficients; $p=4=$ AR model order; ${e}_{k}=$ residual white noise
Cepstrum coefficients	Ceps $i$ (e.g., Ceps1)	${c}_{1}=-{a}_{1}, {c}_{i}=-{a}_{i}-\sum_{l=1}^{i-1}(1-\frac{l}{i}){a}_{n}{c}_{i-1}$ where ${a}_{i}=$ autoregressive coefficients; ${c}_{i}=$ cepstrum coefficients

4.

## Appendix C

See Fig. C1.

To explore the contribution of individual features to the model’s predictions, we applied SHAP (SHapley Additive exPlanations) analysis to the Random Forest classifier trained using the FWD feature set on the full dataset (AIS A to D). We used TreeSHAP, an efficient method specifically designed for tree-based models, to quantify the influence of each feature on the prediction at the level of individual trials.

The SHAP summary plot (Figure C1) indicates that M2 was the most influential feature, with higher values generally contributing towards predictions of positive response. The effects of SSC and MedF are less prominent, and were more variable across samples. This variability may reflect muscle-dependent, non-linear effects that are not easily captured through simple linear interpretation.

Note that, because SHAP explains model output per trial, these results do not directly reflect the final aggregated predictions used in evaluation, which were computed by taking the mode of predicted labels across all trials for each muscle.

## Abbreviations

SCI: Spinal cord injury
UE: Upper extremity
FES: Functional electrical stimulation
EMG: Electromyography
sEMG: Surface EMG
iEMG: Intramuscular EMG
MMT: Manual muscle testing
ISNCSCI: International Standards for Neurological Classification of Spinal Cord Injury
NLI: Neurological level of injury
AIS: American Spinal Injury Association Impairment Scale
NMES: Neuromuscular stimulation
LMN: Lower motor neurons
MEP: Motor evoked potentials
SSEP: Somatosensory evoked potentials
NCS: Nerve conduction studies
MVC: Maximal voluntary contraction
GRASSP: Graded and Redefined Assessment of Strength, Sensibility, and Prehension
MAV: Mean absolute values
DVARV: Difference variance version
M2: Second-order spectral moment
DAMV: Difference absolute mean value
wLen: Waveform length
ZERC: Zero crossings
SSC: Slope sign changes
wAmp: Willison amplitude
logD: Log-detector
ARCO $i$ (e.g., ARCO1): Autoregression coefficients
Ceps $i$ (e.g., Ceps1): Cepstrum coefficients
PCA: Principal component analysis
FWD: Forward selected sEMG features
RF: Random forest
SVM: Support vector machine
GB: Gradient boosting
LOPO: Leave-one-participant-out
MCC: Matthews correlation coefficient
TPR: True positive rate
TNR: True negative rate

## References

[1] Anderson KD. Targeting recovery: priorities of the spinal cord-injured population. J Neurotrauma. 2004;21:1371–83. 15672628 10.1089/neu.2004.21.1371

[2] Lo C, Tran Y, Anderson K, Craig A, Middleton J. Functional priorities in persons with spinal cord injury: using discrete choice experiments to determine preferences. J Neurotrauma. 2016;33:1958–68. 27080545 10.1089/neu.2016.4423

[3] ASIA and ISCoS International Standards Committee. The 2019 revision of the International Standards for Neurological Classification of Spinal Cord Injury (ISNCSCI)—what’s new? Spinal Cord. 2019;57:815–7. 31530900 10.1038/s41393-019-0350-9

[4] Furlan JC, Furlan DT, Marquez-Chin C. A progress report on the spinal cord rehabilitation research initiatives based on registered clinical from 2000 to 2022. Am J Phys Med Rehabil. 2023;102:755–63. 36928768 10.1097/PHM.0000000000002207

[5] Furlan JC, Craven BC. SCI management. Neural repair regen spinal cord inj spine trauma. Elsevier; 2022. p. 349–69.

[6] Marquez-Chin C, Popovic MR. Functional electrical stimulation therapy for restoration of motor function after spinal cord injury and stroke: a review. Biomed Eng OnLine. 2020;19:34. 32448143 10.1186/s12938-020-00773-4 PMC7245767

[7] Popovic MR, Kapadia N, Zivanovic V, Furlan JC, Craven BC, Mcgillivray C. Functional electrical stimulation therapy of voluntary grasping versus only conventional rehabilitation for patients with subacute incomplete tetraplegia: a randomized clinical trial. Neurorehabil Neural Repair. 2011;25:433–42. 21304020 10.1177/1545968310392924

[8] Kapadia NM, Zivanovic V, Furlan J, Craven BC, McGillivray C, Popovic MR. Functional electrical stimulation therapy for grasping in traumatic incomplete spinal cord injury: randomized control trial. Artif Organs. 2011;35:212–6. 21401662 10.1111/j.1525-1594.2011.01216.x

[9] Martin R, Johnston K, Sadowsky C. Neuromuscular electrical stimulation-assisted grasp training and restoration of function in the tetraplegic hand: a case series. Am J Occup Ther. 2012;66:471–7. 22742696 10.5014/ajot.2012.003004

[10] Alon G, McBride K. Persons with C5 or C6 tetraplegia achieve selected functional gains using a neuroprosthesis. Arch Phys Med Rehabil. 2003;84:119–24. 12589632 10.1053/apmr.2003.50073

[11] Kapadia N, Zivanovic V, Popovic MR. Restoring voluntary grasping function in individuals with incomplete chronic spinal cord injury: pilot study. Top Spinal Cord Inj Rehabil. 2013;19:279–87. 24244093 10.1310/sci1904-279 PMC3816722

[12] Ditunno JF, Stover SL, Freed MM, Ahn JH. Motor recovery of the upper extremities in traumatic quadriplegia: a multicenter study. Arch Phys Med Rehabil. 1992;73:431–6. 1580769

[13] Popovic MR, Thrasher TA, Adams ME, Takes V, Zivanovic V, Tonack MI. Functional electrical therapy: retraining grasping in spinal cord injury. Spinal Cord. 2006;44:143–51. 16130018 10.1038/sj.sc.3101822

[14] Coulet B, Allieu Y, Chammas M. Injured metamere and functional surgery of the tetraplegic upper limb. Hand Clin. 2002;18:399–412. 12474592 10.1016/s0749-0712(02)00020-3

[15] Bryden AM, Hoyen HA, Keith MW, Mejia M, Kilgore KL, Nemunaitis GA. Upper extremity assessment in tetraplegia: the importance of differentiating between upper and lower motor neuron paralysis. Arch Phys Med Rehabil. 2016;97:S97-104. 27233597 10.1016/j.apmr.2015.11.021

[16] Bryden A, Kilgore KL, Nemunaitis GA. Advanced assessment of the upper limb in tetraplegia: a three-tiered approach to characterizing paralysis. Top Spinal Cord Inj Rehabil. 2018;24:206–16. 29997424 10.1310/sci2403-206 PMC6037326

[17] Martin R, Sadowsky C, Obst K, Meyer B, McDonald J. Functional electrical stimulation in spinal cord injury: from theory to practice. Top Spinal Cord Inj Rehabil. 2012;18:28–33. 23459150 10.1310/sci1801-28 PMC3584753

[18] Musselman KE, Shah M, Zariffa J. Rehabilitation technologies and interventions for individuals with spinal cord injury: translational potential of current trends. J Neuroeng Rehabil. 2018;15:40. 29769082 10.1186/s12984-018-0386-7 PMC5956557

[19] European Multicenter Study about Spinal Cord Injury [Internet]. http://www.emsci.org/

[20] Noonan VK, Kwon BK, Soril L, Fehlings MG, Hurlbert RJ, Townson A, et al. The Rick Hansen Spinal Cord Injury Registry (RHSCIR): a national patient-registry. Spinal Cord. 2012;50:22–7. 22042297 10.1038/sc.2011.109

[21] Japanese National Spinal Cord Injury Database [Internet]. https://www.kibirihah.johas.go.jp/200_SCI_DB/top.html

[22] Spiess MR, Müller RM, Rupp R, Schuld C, Van Hedel HJA. Conversion in ASIA impairment scale during the first year after traumatic spinal cord injury. J Neurotrauma. 2009;26:2027–36. 19456213 10.1089/neu.2008.0760

[23] Nakajima H, Yokogawa N, Sasagawa T, Ando K, Segi N, Watanabe K, et al. Prognostic factors for cervical spinal cord injury without major bone injury in elderly patients. J Neurotrauma. 2022;39:658–66. 35044252 10.1089/neu.2021.0351 PMC9081051

[24] Håkansson S, Tuci M, Bolliger M, Curt A, Jutzeler CR, Brüningk SC. Data-driven prediction of spinal cord injury recovery: an exploration of current status and future perspectives. Exp Neurol. 2024;380:114913. 39097073 10.1016/j.expneurol.2024.114913

[25] Hubli M, Kramer JLK, Jutzeler CR, Rosner J, Furlan JC, Tansey KE, et al. Application of electrophysiological measures in spinal cord injury clinical trials: a narrative review. Spinal Cord. 2019;57:909–23. 31337870 10.1038/s41393-019-0331-z

[26] Hupp M, Pavese C, Bachmann LM, Koller R, EMSCI Study Group, Schubert M, et al. Electrophysiological multimodal assessments improve outcome prediction in traumatic cervical spinal cord injury. J Neurotrauma. 2018;35:2916–23. 29792368 10.1089/neu.2017.5576

[27] Sherwood AM, Dimitrijevic MR, Barry McKay W. Evidence of subclinical brain influence in clinically complete spinal cord injury: discomplete SCI. J Neurol Sci. 1992;110:90–8. 1506875 10.1016/0022-510x(92)90014-c

[28] Sherwood AM, Graves DE, Priebe MM. Altered motor control and spasticity after spinal cord injury: subjective and objective assessment. J Rehabil Res Dev. 2000;37:41–52. 10847571

[29] Mitchell MD, Yarossi MB, Pierce DN, Garbarini EL, Forrest GF. Reliability of surface EMG as an assessment tool for trunk activity and potential to determine neurorecovery in SCI. Spinal Cord. 2015;53:368–74. 25448189 10.1038/sc.2014.171

[30] Calancie B, Molano MR, Broton JG. EMG for assessing the recovery of voluntary movement after acute spinal cord injury in man. Clin Neurophysiol. 2004;115:1748–59. 15261853 10.1016/j.clinph.2004.03.002

[31] Heald E, Hart R, Kilgore K, Peckham PH. Characterization of volitional electromyographic signals in the lower extremity after motor complete spinal cord injury. Neurorehabil Neural Repair. 2017;31:583–91. 28443786 10.1177/1545968317704904 PMC5560032

[32] Feldner HA, Howell D, Kelly VE, McCoy SW, Steele KM. “Look, Your Muscles Are Firing!”: a qualitative study of clinician perspectives on the use of surface electromyography in neurorehabilitation. Arch Phys Med Rehabil. 2019;100:663–75. 30392855 10.1016/j.apmr.2018.09.120 PMC6435407

[33] Manca A, Cereatti A, Bar-On L, Botter A, Della Croce U, Knaflitz M, et al. A survey on the use and barriers of surface electromyography in neurorehabilitation. Front Neurol. 2020;11:573616. 33123079 10.3389/fneur.2020.573616 PMC7566898

[34] Campanini I, Disselhorst-Klug C, Rymer WZ, Merletti R. Surface EMG in clinical assessment and neurorehabilitation: barriers limiting its use. Front Neurol. 2020;11:934. 32982942 10.3389/fneur.2020.00934 PMC7492208

[35] Merletti R, Campanini I, Rymer WZ, Disselhorst-Klug C. Editorial: Surface electromyography: barriers limiting widespread use of sEMG in clinical assessment and neurorehabilitation. Front Neurol. 2021;12:642257. 33643215 10.3389/fneur.2021.642257 PMC7906963

[36] Balbinot G, Li G, Wiest MJ, Pakosh M, Furlan JC, Kalsi-Ryan S, et al. Properties of the surface electromyogram following traumatic spinal cord injury: a scoping review. J Neuroeng Rehabil. 2021;18:105. 34187509 10.1186/s12984-021-00888-2 PMC8244234

[37] Balbinot G, Wiest MJ, Li G, Pakosh M, Furlan JC, Kalsi-Ryan S, et al. The use of surface EMG in neurorehabilitation following traumatic spinal cord injury: a scoping review. Clin Neurophysiol. 2022;138:61–73. 35364465 10.1016/j.clinph.2022.02.028

[38] Anderson KD, Korupolu R, Musselman KE, Pierce J, Wilson JR, Yozbatiran N, et al. Multi-center, single-blind randomized controlled trial comparing functional electrical stimulation therapy to conventional therapy in incomplete tetraplegia. Front Rehabil Sci. 2022;3:995244. 36188946 10.3389/fresc.2022.995244 PMC9500231

[39] Delagi EF, M. D. Iazzetti J, M. D. Perotto AO, M. D. Morrison D. Anatomical Guide for the Electromyographer: The Limbs and Trunk. 5th edition. Springfield, Ill: Charles C Thomas Pub Ltd; 2011.

[40] Daube JR, Rubin DI. Needle electromyography. Muscle Nerve. 2009;39:244–70. 19145648 10.1002/mus.21180

[41] Roberson DJ, Longbotham HG, Schrader CB. Robust detection of below-lesion, non-invasive electromyographic signals. Biomed Sci Instrum. 1993;29:169–76. 8329587

[42] Hudgins B, Parker P, Scott RN. A new strategy for multifunction myoelectric control. IEEE Trans Biomed Eng. 1993;40:82–94. 8468080 10.1109/10.204774

[43] Zardoshti-Kermani M, Wheeler BC, Badie K, Hashemi RM. EMG feature evaluation for movement control of upper extremity prostheses. IEEE Trans Rehabil Eng. 1995;3:324–33.

[44] Tkach D, Huang H, Kuiken TA. Study of stability of time-domain features for electromyographic pattern recognition. J Neuroeng Rehabil. 2010;7:21. 20492713 10.1186/1743-0003-7-21 PMC2881049

[45] Azhiri RB, Esmaeili M, Nourani M. EMG-based feature extraction and classification for prosthetic hand control. 2021; https://arxiv.org/abs/2107.00733

[46] Phinyomark A, Quaine F, Charbonnier S, Serviere C, Tarpin-Bernard F, Laurillau Y. Feature extraction of the first difference of EMG time series for EMG pattern recognition. Comput Methods Programs Biomed. 2014;117:247–56. 25023536 10.1016/j.cmpb.2014.06.013

[47] Ortiz-Catalan M. Cardinality as a highly descriptive feature in myoelectric pattern recognition for decoding motor volition. Front Neurosci. 2015. 10.3389/fnins.2015.00416. 26578873 10.3389/fnins.2015.00416 PMC4625080

[48] Li G, Balbinot G, Furlan JC, Kalsi-Ryan S, Zariffa J. A computational model of surface electromyography signal alterations after spinal cord injury. J Neural Eng. 2023;20(6):066020. 10.1088/1741-2552/ad0b8e 37948762

[49] Brüningk SC, Bourguignon L, Lukas LP, Maier D, Abel R, Weidner N, et al. Prediction of segmental motor outcomes in traumatic spinal cord injury: advances beyond sum scores. Exp Neurol. 2024;380:114905. 39097076 10.1016/j.expneurol.2024.114905

[50] Buri M, Tanadini LG, Hothorn T, Curt A. Unbiased recursive partitioning enables robust and reliable outcome prediction in acute spinal cord injury. J Neurotrauma. 2022;39:266–76. 33619988 10.1089/neu.2020.7407

[51] Sidon E, Stein M, Ramalingam G, Shemesh S, Benharroch D, Ohana N. Gender differences in spinal injuries: causes and location of injury. J Womens Health. 2018;27:946–51. 10.1089/jwh.2017.6687 29708811

